# Productivity losses and public finance burden attributable to breast cancer in Poland, 2010–2014

**DOI:** 10.1186/s12885-017-3669-7

**Published:** 2017-10-10

**Authors:** Błażej Łyszczarz, Ewelina Nojszewska

**Affiliations:** 1 Department of Public Health, Faculty of Health Sciences, Nicolaus Copernicus University in Toruń, ul, Sandomierska 16, 85-830 Bydgoszcz, Poland; 2 Department of Appiled Economics, Collegium of Management and Finance, Warsaw School of Economics, ul. Madalińskiego 6/8, 02-513 Warszawa, Poland

**Keywords:** Breast cancer, Productivity losses, Indirect costs, Human capital method, Poland, Public finance, Economic burden

## Abstract

**Background:**

Apart from the health and social burden of the disease, breast cancer (BC) has important economic implications for the sick, health system and whole economy. There has been a growing interest in the economic aspects of breast cancer and analyses of the disease costs seem to be the most explored topic. However, the results from these studies are hardly comparable. With this study we aim to contribute to the field by providing estimates of productivity losses and public finance burden attributable to BC in Poland.

**Methods:**

We used retrospective prevalence-based top-down approach to estimate the productivity losses (indirect costs) of BC in Poland in the period 2010–2014. Human capital method (HCM) and societal perspective were used to estimate the costs of: absenteeism of the sick and caregivers, presenteeism of the sick and caregivers, disability, and premature mortality. We also used figures illustrating public finance burden attributable to the disease. Deterministic sensitivity analysis was performed to assess the stability of the estimates. A variety of data sources were used with the social insurance system and Polish National Cancer Registry being the most important ones.

**Results:**

Productivity losses associated with BC in Poland were €583.7 million in 2010 and they increased to €699.7 million in 2014. Throughout the period these costs accounted for 0.162–0.171% of GDP, an equivalent of 62,531–65,816 per capita GDP. Losses attributable to disability and premature mortality proved to be the major cost drivers with 27.6%–30.6% and 22.0%–24.6% of the total costs respectively. The costs due to caregivers’ presenteeism were negligible (0.1% of total costs). Public finance expenditure for social insurance benefits to BC sufferers ranged from €50.2 million (2010) to €56.6 million (2014), an equivalent of 0.72–0.79% of expenditures for all diseases. Potential losses in public finance revenues accounted for €173.9 million in 2010 and €211.0 million in 2014. Sensitivity analysis showed that the results were robust to changes in the model parameters.

**Conclusions:**

The productivity losses attributable to BC in Poland were a sizable burden for the society. They contributed both to decreased economy output and to public finance deficit.

## Background

Similarly to virtually all developed countries breast cancer (BC) is one of the major health problems in Poland. It is the most frequently diagnosed cancer in Polish women; with 17,379 cases in 2014 it accounted for 22% of oncological diagnoses among females [1]. The increasing trend in BC is observed both in terms of incidence and mortality; between 2010 and 2014 these (standardised) measures grew by around 4.3 and 7.5% respectively [2]. With the incidence rate of 69.9 per 100,000 women in 2012 Poland located notably below the European mean value (92.8); also, the mortality rates were relatively low there and accounted for 19.7 deaths per 100,000 population, 3.4 less than on average in Europe [3]. Despite this fairly favourable epidemiological situation these women who develop BC in Poland have less chance to survive; 1-year relative survival rate for Poland is 90.9% which is almost 4 percentage points (p.p.) lower than in Europe (94.8%) and the gap rises to more than 10 p.p. for 5-year survival rate (71.6 and 81.8% respectively) [4].

Apart from the health and social burden of the disease, BC has important economic implications for the sick, health system and whole economy, including public finance. High incidence of the condition and dynamic improvement in its treatment result in substantial expenses for BC care, both private and public. The results from Poland show that the average patients’ out-of-pocket expenses for treating advanced BC in 2013 accounted for 850 zlotys per month (an equivalent of €202.5), around 23% of average remuneration [5]. Moreover, the mean public expenditure for treating a BC patient increased by 55% between 2004 and 2010 exceeding the inflation rate in the same period threefold [6]. From a broader economic perspective, BC is more often diagnosed among women at working age; the incidence of the disease in females aged 20–59 increased from 56.8 per 100,000 women in 1999 to 67.8 per 100,000 women in 2014, resulting in potentially higher productivity losses due to the illness [2].

The economic aspects of BC have been subject to growing awareness in health services research and most studies focused on costs of the disease. The studies estimating direct costs conducted in the United States [7–11], Germany [12], Poland [6, 13] and Lithuania [14] provide evidence on the magnitude of costs associated with BC treatment while the research focusing on indirect costs estimate productivity losses in Spain [15] and Lithuania [16]. Recently, there has been a growing interest in research combining direct and indirect costs within the cost-of-illness framework [17] as examples from Iran [18], Korea [19], Flanders [20], Sweden [21], Japan [22] and California [23] show. Despite this relative abundance of the evidence on the BC costs in various countries there are still gaps in our knowledge on the economic consequences of the disease. This results from the fact that the research from various countries differ notably in terms of the cost categories included and the estimation methods leading to hardly comparable results. For example, the study from Sweden [21] is the only one that comprises intangible costs, such as pain and suffering. Also, the indirect costs estimation differs notably, with the Californian research [23] focusing solely on mortality costs, Iranian estimates [18] including productivity losses of caregivers absenteeism among others and none of the studies estimating losses due to presenteeism, either of the sick or their relatives.

These methodological differences lead to different cost estimates in particular national or regional settings and do not allow to draw comparable conclusions on the economic burden that societies face due to BC. The aim of this paper is to contribute to a growing body of evidence on the costs of BC by the estimating productivity losses and public finance burden associated with the disease in Poland. To begin with, it is the first study which tries to estimate overall indirect costs, including the losses attributable not only to absenteeism and premature mortality of the sick, but also to caregivers’ absenteeism and to the decreased productivity (presenteeism) of both the sick and their caregivers. Secondly, we supplement the usual approach to estimate economic burden of the disease by analyzing its consequences for public finance. Public agents nowadays spend significant proportions of their budgets for sickness benefits and allowances; moreover, a part of potential tax revenues are lost because of gross domestic product (GDP) unproduced due to the illness. This study is the first one that attempts to estimate both kinds of these public finance losses. Finally, we formulate some recommendations to increase comparability of the results for future studies.

## Methods

### General assumptions

This study uses retrospective prevalence-based top-down approach to estimate the productivity losses (indirect costs) due to BC in Poland in the period 2010–2014. Human capital method and societal perspective were used to estimate the costs of the following components of economic inactivity:

- absenteeism of the sick;
- presenteeism of the sick;
- informal caregivers’ absenteeism;
- informal caregivers’ presenteeism;
- premature mortality caused by the disease;
- disability caused by the disease.

The cost of housekeeping activities was not included in the analysis because this category of costs presents several challenges in estimation and a lack of specific data for Poland prevents us from including it into our analysis.

Mean GDP per worker was used as a measure of labour productivity. Unlike in previous studies, in our estimates we accounted for decreasing marginal productivity of labour. This assumption in economic modelling means that each incremental employee in an economy produces decreasing increment of the output. For this reason, the output increments that would have been gained in the absence of the disease would be lower for each additional employee as compared to average productivity in the economy. Therefore, using mean GDP per worker overestimates the real magnitude of productivity losses attributable to the disease. To account for decreasing marginal productivity we follow the recommendations for indirect cost estimation methodology in Poland [24] and use correction coefficient of 0.65; this value reflects a relationship between marginal and average labour productivity and it approximates output elasticity of labour in Cobb-Douglas production function as used by European Commission in calculating potential growth rates [25].

Table 1 provides a description of the main parameters used in the estimation of productivity losses due to BC.

**Table 1:** Main parameters of model for estimating productivity losses associated with breast cancer in Poland

	Parameter (unit)	Mean value for years 2010–2014
General economic parameters		
Gross domestic product (€)		387,583,804 353^1^
Per worker gross domestic product (€)		27 126^1^
Correction coefficient to adjust for decreasing marginal labour productivity		0.65
Exchange rate (zlotys per €)		4.14
Parameters for estimating indirect costs		
Absenteeism of the sick	Number of absence days	1121 107^2^
	Number of people receiving first-time rehabilitation benefits	2103
	Average duration of first-time rehabilitation benefits (months)	7.62
	Number of people receiving renewed rehabilitation benefits	755
	Average duration of renewed rehabilitation benefits (months)	5.26
Presenteeism of the sick	Number of the sick people (5-year prevalence)	68 126^3^
	Employment rate of women at age 25–59	67%
	Rate of productivity reduction while working	29,8%^4^
Caregivers’ absenteeism	Number of absence days due to a relative’s illness	5817
Caregivers’ presenteeism	Share of employed population that provides informal care to an oncological patient	0.48%^5^
	Number of people who work and provide care for BC patients	34,324
	Rate of caregivers’ productivity reduction while working	21%^6^
Premature mortality	Number of deaths at age 18–59	1707
	Retirement age for women (years)	60
	Economy’s yearly productivity growth for period 2015–2049^7^	2.3%
Disability	Number of people receiving disability pensions^2,8^	
	• permanent pension • temporary pension	569 4872
	Average duration of temporary disability pension in all cancers (months)	18.5
	1-year BC survival rate [4]	90.9%

Notes: Unless stated otherwise, all values refer to yearly mean for period 2010–2014; 1 – values in Euro currency (€) calculated using constant average 2010–2014 exchange rate: 4.14 zlotys per €; 2 – real data is used for population insured in the Social Insurance Institution; for those insured in the Agricultural Social Insurance Fund the data is estimated; 3 – real value for year 2012 [30]; for other years the value was estimated; 4 – the average value based on [31–33]; 5 – due to a lack of BC-specific data, the rate refers to caregivers in all cancers in Poland [34] and the share of those with BC in total cancer patients is used to estimate the number of working caregivers for BC patients; 6 – due to an unavailability of BC-specific productivity reduction of caregivers, data for all cancers in Poland is used [34]; 7 – the timespan covers the period of potential economic activity of the youngest women who develop BC during the period investigated; based on [35]; 8 – the values show an equivalent of people who are completely unable to work assuming that a partial inability to work corresponds to 0.75 of complete inability to work

We used several sources of data to estimate the indirect costs borne by the Polish society due to BC. In the following subsections we describe the methodological approach used to estimate the costs and give details on the data sources used.

### Absenteeism

Absenteeism refers to a temporary absence from work due to illness. The scale of absenteeism is usually identified through surveys conducted among a sample of patients or by using administrative data. Here, we used data published by the Social Insurance Institution (*Zakład Ubezpieczeń Społecznych* - ZUS) [26] and received from the Agricultural Social Insurance Fund (*Kasa Rolniczego Ubezpieczenia Społecznego* - KRUS), two institutions that operate social benefits payments for general population and farmers, respectively. In Poland, an absence lasting up to 180 days is subject to sick allowance while in the case of prolonging inability to work (but with a predicted recovery that would allow returning to work) a rehabilitation benefit lasting up to another 12 months is issued. Each sickness episode of an employed person is reported to ZUS or KRUS through a medical certificate issued by a physician; the certificates contain data on ICD-10 code which allows for identifying those absence days that can be assigned to BC. The magnitude of short-term absence in general (non-farmers) population can be identified because ZUS reports exact numbers of absent days due to each specific ICD-10 code. For farmers we were only able to obtain data on total absence days in a given year with no information on disease-specific absence; thus, we assumed that the share of absence days due to BC in farmers population was the same as in those insured in ZUS. The losses due to absence lasting longer than 180 days were estimated solely with ZUS data (farmers’ insurance fund does not grant rehabilitation benefits) based on the number of rehabilitation benefits and the average time for which first-time and renewed benefits were issued.

Summing up the duration of short-term sick allowances and rehabilitation benefits reported by ZUS and KRUS we obtained an estimate of time lost due to short- and medium-term work inactivity caused by BC. The product of years lost due to illness and per worker GDP adjusted for 0.65 correction coefficient makes up the cost of BC absenteeism in Poland.

### Presenteeism

Presenteeism refers to a situation in which sick people continue to work, though, their productivity is decreased due to illness. Because BC is considered to be a chronic illness, a part of those experiencing the disease continue to work [27, 28] but their productivity is lower than in the absence of the disease. The identification of presenteeism’s magnitude is typically more challenging than in the case of absenteeism; however, with increasing evidence on health-related quality of life and labour participation in cancer, we are now able to estimate these societal losses. The first step was to identify the number of individuals with BC; to approximate this number we used 5-year prevalence of the disease as suggested in literature on cancer epidemiology [29]. Because the prevalence measure is not reported on yearly basis, we used its value for 2012 [30] and estimated the numbers for remaining years using mean value of two ratios: incidence to 5-year prevalence, and mortality to 5-year prevalence. In the next step we proxied the number of those with BC being at a productive age (15–59 years^1^) and adjusted it for women employment rate. From this amount we subtracted the numbers of newly granted disability pensions and rehabilitation benefits to exclude those who were not working due to disability. Next, to account for absenteeism, we subtracted the number of sick leave days due to BC from the number of working days in each year. In this way, we obtained the number of working days of those with BC who remained active in the labour market.

The extent to which a sick person’s productivity is decreased because of BC has not been investigated in Poland so far. Thus, relying on three studies from the Netherlands and Sweden [31], the United States [32] and Japan [33] which deal with presenteeism in BC, we used a mean value of 29.8% productivity decrease due to this condition. The product of decreased productivity, number of days worked by those with BC and daily per worker GDP corrected with 0.65 coefficient yields the cost of presenteeism.

### Informal caregivers’ absenteeism

Indirect costs are not limited solely to lost or lower productivity of the sick. In the case of severe health deterioration which prevents a sick person from functioning independently and gives a reason for providing care by a third party individual we encounter the caregiver’s lost productivity. The situation when a caretaker temporarily suspends work is called the caregiver’s absenteeism. The magnitude of this component of indirect costs depends on the specificity of the disease; e.g. childhood diseases and conditions that severely limit mobility of the sick are the ones that require more attention from caretakers and generate more losses of productivity.

The cost of informal caregivers’ absenteeism was estimated by using social insurance data. In Poland, a person who provides informal care for either a child or other relative receives care allowance and this fact is reported by ZUS. In our estimation we only included data on care provided for adults because children hardly ever experience BC (in 2010–14 there was one case of the disease in 0–19 years age group in Poland). ZUS collects data only on the number of care days with no disease-specific information; thus, to approximate the care days related to BC we assumed that the share of medical certificates issued for BC caregivers is the same as for the certificates related to own sickness for which disease-specific data was obtainable. The number of work days lost was multiplied by daily per worker GDP and corrected with 0.65 coefficient yielding indirect cost of BC caregivers’ absenteeism.

### Informal caregivers’ presenteeism

The care provided to a sick person not only diminishes informal caregivers’ labour supply, it also may affect their productivity. Physical and mental burden experienced by carers might result in their lower efficiency at work. This component of indirect costs is potentially more meaningful in chronic diseases in which caregivers contribute to care through longer periods of time, experience cumulative fatigue and, as a consequence, work with decreased productivity. We began our assessment of caregivers’ presenteeism with estimating the number of people who work and are engaged in providing care to a family member suffering from BC. The results of the survey representative for the Polish population conducted in years 2011–12 show that 0.48% of those working provide care for their relatives with cancer [34]. The product of this share and the population of Poland yielded the number of cancer caregivers. From this number we approximated the number of BC carers assuming that the proportion of BC carers to all cancer carers is the same as the proportion of BC sufferers to all people with cancer (we used 5-years prevalence as a measure of people with cancer). In the next step we calculated the amount of BC caregivers’ working days and subtracted the number of caregivers’ absenteeism days to obtain the number of days that carers worked with diminished productivity. Because we have not found any research on the magnitude of carers’ productivity decline in BC we used the value of 21% decline estimated for those providing informal care for all cancers in Poland [34]. Finally, the productivity loss due to BC caregivers’ presenteeism was calculated as a product of the value of GDP produced by caregivers, the 21% decline of productivity and the 0.65 correction coefficient.

### Premature mortality

Premature mortality is a component of indirect costs because deaths of people at working age decrease an economy’s potential output. Regarding the context of this study, we define premature death as the one that occurs before retirement age. Using HCM, the production lost due to premature deaths was estimated as a discounted value of output that would be produced if those who died prematurely were still alive and were working until their retirement age.

We used mortality rates due to BC in 5-year age groups and assumed that the distribution of deaths within each group was the same as in the total women mortality in Poland. In this way, we obtained the number of deaths at every age from 19 until 60 which is a retirement age for women in Poland. To account for other than BC causes of mortality and for the fact that not all patients who died would work in the future, we adjusted the number of deaths for age-specific survival probability and for employment rate among women at age 25–59. The value of economic output lost due to the death of those identified in the above way was estimated by summing the products of the number of deaths at each employment age and the age-specific discounted value (5% discount rate was used [35]) of potential production lost for every age from 19 until the retirement age. The result was corrected by 0.65 as in each other cost component. The values of future GDP were based on forecasted productivity growth of the Polish economy as projected by European Commission [36].

### Disability

In this study, disability refers to long-term or permanent inability to work due to a health condition. The mechanism behind productivity losses due to disability caused by BC is the same as the one in absenteeism; though, we distinguish these two components to provide a more comprehensive view on the structure of indirect costs related to the condition.

In estimating disability costs we relied on data from the social insurance system. Both ZUS and KRUS grant disability pensions for those who are unable to work due to disease or accidents at work. Doctors working for social insurers evaluate incapacity to work, its degree (complete or partial incapacity to work) as well as permanency or expected duration of the incapacity and issue a certificate which entitles a person to disability pension. There are four categories of these pensions: (1) permanent and complete; (2) permanent and partial; (3) temporary and complete; (4) temporary and partial inability to work. We had to make several assumptions and adjustments to approximate this category of costs. First, a person partially unable to work produces 1/4 of the average output of a healthy worker.^2^ Second, the average time of temporary inability to work was 17.8–19.3 months depending on the year, which was a value for all cancers, not BC. Third, to avoid double counting we adjusted the number of the disabled for 1-year survival rate for BC in Poland [4] and for other than BC causes of death for those at 56–58 years of age, which was the mean age of women receiving disability pension in Poland in 2010–2014. For each of the four pension categories we estimated the number of people receiving benefits, the average time of pension duration and the discounted value of production loss corresponding to each category. Summing up the losses identified for all these categories and correcting for 0.65 coefficient we approximated the indirect cost of disability caused by BC.

### Public finance spending and potential budget revenue losses

In order to identify the consequences of BC for public finance in Poland we estimated (1) the social security system’s expenditure attributable to the disease and (2) potential public revenues lost resulting from the economy’s decreased output. The data to calculate (1) was obtained from SSI which operates sickness insurance system in Poland. To estimate (2) we calculated the shares of four main taxes (personal income tax; corporate income tax; VAT and excise tax^3^) and social insurance premiums in annual GDP and multiplied these shares by GDP lost due to BC; the product shows a potential revenue loss in the state and regional budgets and social insurance funds as a result of the disease.

### Sensitivity analysis

Deterministic one-way sensitivity analysis was performed to assess the impact of changes in the key model parameters on the productivity losses estimates. To test the stability of the results we used: 0% and 3.5% discount rates; extreme exchange rates from the period analysed (3.99–4.20 zlotys per €) instead of the average rate; values of 0.6 and 0.7 for correction coefficient which adjusts results for decreasing marginal labour productivity; varying values of productivity reduction rate in presenteeism of the sick (range from 21% to 34%) and caregivers’ presenteeism (range from 15.4 to 21.5%) according to the estimates found in literature [31, 33, 37, 38]; ±20% variation in number of caregivers’ absence days; and gross value added instead of GDP as a productivity measure.

## Results

### Epidemiological trends

The number of BC cases diagnosed among women and men in Poland raised from 15,891 in 2010 to 17,506 in 2014, a 10.2% increase over the 4-year period. The highest rate of increase was observed among those at their 60s (27.6%), followed by the youngest (0–39 years: 17.9%) and the oldest (≥70 years: 17.8%) groups. The incidence decreased only in the population at their 50s (−10.9%). The standardises incidence rate in total women population was 67.1 per 100,000 population in 2010 and it increased to 70.0 four years later. In terms of incidence rate dynamics we observed the highest increase for the oldest (≥70 years: 17.5%) and the youngest (0–39 years: 10.0%) women (Table 2).

**Table 2:** Age distribution of breast cancer incidence and deaths in Poland in 2010–2014

	Number of diagnosed breast cancer cases (standardises incidence rate - per 100,000 women)						Number of deaths from breast cancer (standardises mortality rate - per 100,000 women)					
Age group (years)	2010	2011	2012	2013	2014	Change 2010/2014	2010	2011	2012	2013	2014	Change 2010/2014
0–39	788 (6.80)	863 (7.30)	895 (7.42)	901 (7.30)	929 (7.48)	17.9% (10.0%)	106 (0.92)	116 (0.98)	126 (1.04)	137 (1.12)	140 (1.12)	32.1% (21.7%)
40–49	2092 (85.00)	2192 (90.58)	2175 (90.31)	2212 (91.83)	2232 (92.05)	6.7% (8.3%)	424 (17.10)	446 (18.28)	360 (14.93)	378 (15.66)	412 (17.05)	−2.8% (−0.3%)
50–59	4935 (161.66)	4940 (163.53)	4841 (163.06)	4651 (159.32)	4398 (155.03)	−10.9% (−4.1%)	1195 (38.78)	1169 (38.27)	1182 (39.04)	1236 (41.66)	1134 (39.28)	−5.1% (1.3%)
60–69	4399 (220.25)	4909 (235.51)	5121 (230.58)	5433 (231.42)	5615 (226.23)	27.6% (2.7%)	1173 (58.68)	1329 (63.26)	1459 (65.39)	1518 (64.61)	1641 (66.35)	39.9% (13.1%)
≥ 70	3677 (149.93)	3739 (151.18)	4112 (164.79)	4089 (165.76)	4332 (176.19)	17.8% (17.5%)	2387 (92.92)	2437 (93.64)	2524 (95.77)	2612 (98.28)	2697 (102.15)	13.0% (9.9%)
Total	15,891 (67.11)	16,643 (69.88)	17,144 (70.36)	17,286 (70.16)	17,506 (70.00)	10.2% (4.3%)	5285 (19.75)	5497 (20.34)	5651 (20.35)	5881 (20.94)	6024 (21.25)	14.0% (7.6%)

Source: [2]. Notes: Standardised incidence rates are calculated using the European population as a standard population. The Number of diagnosed cases and deaths refers to both men and women, while the rates (in parentheses) refer to women solely

The number of deaths from BC in Poland raised from 5285 to 6024 in the period investigated (14% increase). The rise was mostly due to a dynamic increase in 60–69 years population (from 1173 to 1641 deaths; 39.9%); however, in terms of relative changes, also the youngest group experienced a striking growth of deaths with a 32.1% change. On the other hand, the total mortality declined among those at 40s and 50s. The standardised mortality rate increased by 7.6%, from 19.75 in 2010 to 21.25 in 2014 and the youngest were those where the increase in the rate was the highest (21.7%) and the only group with a decreasing rate was those at 40–49 years (Table 2).

### Productivity losses

The productivity losses due to BC in Poland in 2010 were estimated at €583.7 million and they increased to €699.7 million in 2014, exhibiting a 20% increase over the period. The highest loss in each year was attributable to disability (€178.0 million to €204.2 million) followed by premature mortality (€139.8 million to €167.0 million) and caregivers’ presenteeism (€116.0 million to €133.5 million). The importance of burden caused by caregivers’ absenteeism in BC was marginal with only €0.3–€0.5 million loss (Table 3).

**Table 3:** Productivity losses associated with breast cancer in Poland in 2010–2014

		Absenteism of the sick	Presenteeism of the sick	Caregivers’ absenteeism	Caregivers’ presenteeism	Premature mortality	Disability	Total
2010	Total cost (€)	86,973,338	62,569,095	336,886	116,000,973	139,794,844	177,993,508	583,668,644
	% of GDP	0.0249	0.0179	0.0001	0.0332	0.0400	0.0509	0.1670
	Times per capita GDP	9592	6901	37	12,794	15,418	19,631	64,373
2011	Total cost (€)	98,865,487	69,755,233	383,471	124,666,405	151,323,562	169,511,133	614,505,291
	% of GDP	0.0261	0.0184	0.0001	0.0329	0.0400	0.0448	0.1623
	Times per capita GDP	10,060	7098	39	12,686	15,399	17,249	62,531
2012	Total cost (€)	111,628,271	69,209,888	412,542	130,169,521	143,704,020	196,958,492	652,082,734
	% of GDP	0.0283	0.0176	0.0001	0.0331	0.0365	0.0500	0.1656
	Times per capita GDP	10,922	6772	40	12,737	14,061	19,272	63,804
2013	Total cost (€)	117,544,495	68,114,403	429,957	131,551,900	157,278,835	209,702,585	684 622,175
	% of GDP	0.0294	0.0170	0.0001	0.0329	0.0393	0.0524	0.1711
	Times per capita GDP	11,300	6548	41	12,647	15,120	20,160	65,816
2014	Total cost (€)	127,402,664	67,121,428	485,410	133,474,886	167,044,901	204,185,099	699,714,388
	% of GDP	0.0307	0.0162	0.0001	0.0321	0.0402	0.0491	0.1684
	Times per capita GDP	11,796	6215	45	12,358	15,466	18,905	64,785

Source: own estimates. Notes: Total cost values in Euro currency calculated using the constant average 2010–2014 exchange rate: 4.14 zlotys per €

To account for economy’s growth we expressed the losses in relation to GDP. This approach shows that the magnitude of the productivity losses caused by BC was relatively stable across the period. In 2010 these losses approximated to 0.1670% of GDP and 64,373 per capita GDP while in 2014 the respective values increased to 0.1684% and 64,785. However, when using GDP-related values we did not observe increases in each consecutive year; in 2011 and 2014 the year-to-year losses declined (Table 3).

**Fig. 1:**
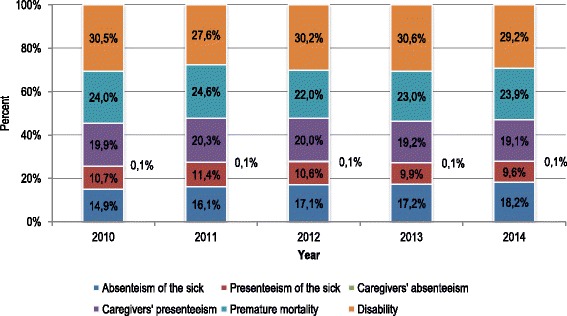
Structure of productivity losses categories in breast cancer in Poland, 2010–2014. Source: own calculations. Notes: The value of 0.1% at the right from each bar refers to caregivers’ absenteeism which is too low to be readable directly from the figure

Of the six indirect cost categories, productivity losses associated with disability were the highest and ranged from 27.6% to 30.6% of the total costs depending on year. Losses due to premature mortality amounted to 22.0%–24.6% of the total burden, caregivers’ presenteeism constituted around one-fifth of the costs, magnitude of the sick’s absenteeism ranged from 14.9% to 18.2% of the total productivity losses and presenteeism of the sick was responsible for 9.6%–11.4% of the indirect costs in BC. Magnitude of caregivers’ presenteeism was very low with only 0.1% of these costs (Fig. 1).

**Fig. 2:**
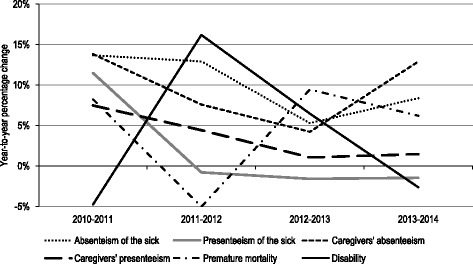
Dynamics of productivity losses categories in breast cancer in Poland, 2010–2014. Source: own calculations

The dynamics of the six indirect costs components exhibits varied patterns of development. Absenteeism of both the sick and caregivers as well as carers’ presenteeism increased in each year (comparing to the previous year), while in the other three categories (presenteeism of the sick, premature mortality and disability) we observed at least 1 year with declining productivity losses (Fig. 2).

### Public finance burden

The expenditure of the Social Insurance Institution for benefits related to breast cancer in Poland was €50.2 million in 2010 and it increased to €56.6 million in 2014. The values corresponded to around 14.8–15.2% of the ZUS’s expenditure for all cancers (C00-D48) and 0.72–0.79% of the ZUS’s expenditure for all diseases (A00-Z99) depending on year. A majority of these expenditures was related to disability pensions; however, the amounts spent on this benefit category decreased from €35.6 million in 2010 to €30.5 million in 2014. On the other hand, the amounts spent on sickness benefits and rehabilitation benefits increased during the period investigated. The spending on medical rehabilitation within the framework of disability prevention and social pensions^4^ were low, but they were increasing rapidly during the period (Table 4).

**Table 4:** Social insurance expenditures for benefits associated with breast cancer in Poland in 2010–2014 (€)

	2010	2012	2013	2014
Rehabilitation benefits	4,006,074	8,864,563	9,627,937	10,772,012
Medical rehabilitation within the framework of disability prevention	238,633	433,060	619,935	767,528
Disability pensions	35,560,519	34,247,032	37,474,105	30,458,545
Social pension	42,711	75,913	89,773	196,297
Sickness benefits	10,337,799	12,470,610	13,702,034	14,452,821
Total expenditures for BC benefits (% of expenditures for all diseases)	50,185,736 (0.72)	56,091,179 (0.76)	61,513,784 (0.79)	56,647,203 (0.72)
Total expenditures for all cancers (ICD-10 codes: C00-D48) benefits	340,041,084	369,594,098	404,581,868	373,612,734
Total expenditures for all diseases (ICD-10 codes: A00-Z99) benefits	6,928,981,365	7,358,678,780	7,802,910,679	7,866,663,531

Notes: Data for 2011 was not available. Data refers only to the Social Insurance Institution’s expenditure. Data for the Agricultural Social Insurance Fund was not obtainable. All values in Euro currency calculated using the constant average 2010–2014 exchange rate: 4.14 zlotys per €

To account for a potential reduction of public revenues due to the productivity losses attributable to BC we calculated shares of taxes and social insurance contributions in GDP and multiplied these shares by the indirect costs estimated. We used data for VAT, excise tax, personal and corporate income taxes (PIT and CIT) as well as social and health insurance contributions which together constituted around 30% of GDP in Poland in the period under consideration (Table 5, panel A). The total potential public revenue losses due to BC were €173.9 million in 2010 and they increased to €211.0 million 4 years later. Among taxes, losses due to VAT and PIT revenues reduction were the highest, with the 2014 values of €49.2 million and €31.5 million, respectively. The potential losses in social insurance contributions increased considerably from 69.9 million in 2010 to 92.7 million in 2014 (Table 5, panel B).

**Table 5:** Potential losses in public revenues due to breast cancer in Poland in 2010–2014

	A: Share of revenues from taxes and social insurance contributions as a proportion of GDP^a^ (%)					B: Public finance revenue losses due to BC^b^ (€)				
	2010	2011	2012	2013	2014	2010	2011	2012	2013	2014
VAT	7.49	7.52	7.31	7.15	7.04	43,739 312	46,180,545	47,660,486	48,932,271	49,243,999
Excise tax	3.84	3.75	3.69	3.65	3.62	22,399,909	23,073,416	24,067,985	24,998,536	25,341,655
Corporate income tax	2.07	1.97	1.92	1.82	1.75	12,093,301	12,120,643	12,521,393	12,466,206	12,250,431
Personal income tax	4.41	4.32	4.37	4.44	4.50	25,759,900	26,564,313	28,468,010	30,426,104	31,477,840
Social insurance contributions. Incl.	11.97	12.24	12.75	13.14	13.25	69,869,282	75,236,103	83,125,732	89,959,727	92,693,135
- health insurance contributions	3.83	3.75	3.72	3.73	3.75	22,334,633	23,016,703	24,275,523	25,550,569	26,217,509
Total	29.79	29.81	30.03	30.20	30.16	173,861,704	183,175,019	195,843,606	206,782,845	211,007,060

Notes: ^a^a moving average for 3 years was used for each year in order to account for possible unusual fluctuations in a particular year; for 2014 – a moving average for years 2013 and 2014; ^b^values in Euro currency calculated using the constant average 2010–2014 exchange rate: 4.14 zlotys per €

### Sensitivity analysis

Table 6 reports the results of one-way sensitivity analysis for the productivity losses estimates. For the sake of brevity, we restricted the analysis to year 2014 solely. Using 3.5% discount rate increased the estimates only vaguely (3.4%); with no costs discounting the total productivity losses were 14.1% higher than in the base scenario. Variation in the exchange rate as well as the rate of productivity reduction in presenteeism of both the sick and caregivers had little effect on the losses estimated. Variation in caregivers’ absenteeism resulted in unnoticeable changes in estimates. Changing the value of correction coefficient by ±0.05 led to a 7.7% change in the indirect costs. The lowest estimates in the sensitivity analysis were obtained with gross value added used as a productivity measure resulting in 11.3% lower estimates compared to the base scenario.

**Table 6:** Sensitivity analysis for productivity losses due to BC in Poland (2014) according to varying assumptions regarding model parameters

	Total productivity losses (€)	Change from base scenario
Base scenario (BS)	699,714,388	–
Discount rate (BS: 5%)		
0%	798,564,742	14.1%
3.5%	723,264,686	3.4%
Exchange rate (BS: 4.14 zlotys per €)		
3.99	724,556,298	3.6%
4.20	689,532,481	−1.5%
Coefficient to adjust for decreasing marginal labour productivity (BS: 0.65)		
0.6	645,890,204	−7.7%
0.7	753,538,571	7.7%
Rate of productivity reduction for presenteeism of the sick (BS: 29.8%)		
21%	679,893,295	−2.8%
34%	709,174,455	1.4%
Number of caregivers’ absence days (BS: 6542)		
5234 (−20%)	699,617,306	0.0%
7850 (+20%)	699,811,470	0.0%
Rate of productivity reduction for caregivers’ presenteeism (BS: 21%)		
15.4%	664,121,085	−5.1%
21.5%	702,892,361	0.5%
Productivity measure (BS: Gross domestic product)		
Gross value added	620,790,733	−11.3%

Source: own estimates

## Discussion

This study on the economic aspects of BC estimated productivity losses and public finance burden attributable to the disease in Poland in the period 2010–2014. For that purpose we used the retrospective prevalence-based top-down approach and data from a variety of sources (mainly from social insurance information system and national cancer statistics). This is the first study on indirect costs of BC which attempted to estimate overall indirect costs, including not only absenteeism of the sick and mortality costs but also losses attributable to presenteeism of the sick as well as caregivers’ absenteeism and presenteeism. This approach resulted in obtaining more comprehensive estimates of productivity losses attributable to BC which are closer to identifying the real economic burden experienced by a society than the results from previous studies. The other contribution of this paper was to identify the scope of losses caused by BC in terms of public finance burden.

The results show that the productivity losses (indirect costs) associated with BC in Poland were €583.7 million in 2010 and grew to €699.7 million in 2014, a 20% increase. However, when accounting for economic growth by expressing these costs in relation to GDP, economic burden is stable over time; in 2010 losses accounted for 0.167% of GDP (64,373 per capita GDP) while 4 years later they constituted 0.168% of GDP (64,785 per capita GDP). This shows that despite the changing epidemiological patterns of BC in Poland (growing incidence and mortality among younger groups) productivity losses remained fairly unchanged during the 5-year period. Of the six indirect costs categories, losses due to disability, premature mortality and caregivers’ presenteeism caused the highest economic burden, accounting for 29.6, 23.5 and 19.7% (average values for the whole period) of total costs respectively. Throughout the period analysed the magnitude of costs associated with absenteeism of the sick grew gradually; they amounted to 14.9% of the total costs in 2010 and reached the share of 18.2% in 2014. The results also show that the losses due to carers’ presenteeism in BC are negligible (0.1% of total costs).

Considering the public finance burden caused by BC we identified 12.9% increase in social insurance expenditure during the period (from €50.2 million in 2010 to €56.6 million in 2014), considerably lower than the increase of indirect costs. Interestingly, the structure of social benefits paid to BC patients changed over the period; the expenditure for disability pensions decreased by 14% while the spending for sickness and rehabilitation benefits increased by 40% and 169% respectively between 2010 and 2014. These contrasting tendencies illustrate a decreasing magnitude of long-term benefits and growing importance of short- and medium-term benefits. There are at least two possible explanations for this tendency. Firstly, recent advances in treatment and rehabilitation allow BC survivors to return to work after a shorter period of time. Secondly, the social insurance policy in Poland is recently aimed at limiting the number of long-time disability benefits and encouraging those unable to work to recover and return to labour force [39] as illustrated by increased amounts paid to sickness and rehabilitation benefits. Considering the potential lost public funds’ revenues due to BC we observed a 21.4% increase (from €173.9 million to €211.0 million) between 2010 and 2014 indicating a significant growth of losses for public revenues.

The sensitivity analysis conducted shows that our estimates are robust to changes in the model parameters. With no discounting the productivity losses were 14.1% higher than in the base scenario and this variation was the highest among all the assumptions tested. The relatively low impact of 0% discount rate results from the fact that a majority of cases in BC to which discounting applies (deaths and disability) occur in later periods of life and in this circumstance the discounting effect is limited to a few periods. On the other hand, the lowest estimates obtained were 11.3% lower than in the base scenario and they effected from using gross value added as a productivity measure. Given that all other changes in the model parameters resulted in less than 10% changes in the indirect costs estimated, we conclude that our findings are fairly stable.

Numerous studies have reported on productivity costs attributable to BC. One previous study provided the estimates of indirect costs for Poland [34]; however, the results reported there are not directly comparable to ours. According to the results from 2009 breast cancer generated productivity losses of 1.17 billion zlotys (€283.6 million using the exchange rate from our study) and accounted for 10% of indirect costs associated to all cancers. The study used gross value added as a measure of employee’s productivity, it also did not account for presenteeism and both these facts make the costs identified lower than these from our study. The study from Lithuania, Poland’s neighbouring country, provides an estimate of €56 million of BC indirect costs in 2008 [16]. Again, this result is hardly comparable to our estimate because the costs from Lithuania include budget expenditure for disability allowances and pensions, a rather uncommon approach in estimating indirect costs. The study from Japan estimated the costs of BC morbidity and mortality in 2011 for US$5.31 billion and showed that the increase of these costs from 1996 to 2011 was significant (a 3.8% annual growth rate) while it was predicted that until 2020 the growth of the costs would decline to 0.7% annually [22]. The estimates for the indirect costs of BC in 2001 in California accounted for mortality solely and identified economic burden due to this reason as US$1.15 billion [23]. A recent study from Korea shows that during 4-year period (2007–2010) the indirect costs of BC increased by 37.3% (from US$339 million to US$465 million), significantly more than in our study [19]. Estimates from Spain illustrate how the results of indirect costs in BC differ with the methodological approach chosen. Using human capital approach, similarly to our study and the other ones discussed above, the indirect cost of BC in Spain in 2003 was €288.7 million while with friction costs approach it was only €11.6 million [15].

The variety of methodological approaches used in the studies discussed makes comparisons of results difficult. These difficulties arise from a number of reasons, of which data availability and comparability in a particular regional and national settings seem to be the most challenging. Principally, there is no uniform, widely agreed system of data collection for the purpose of indirect costs estimation that would allow for including the same cost categories in different settings. Also, there is no agreement on issues like the method of productivity costs estimation (human capital approach vs. friction costs method); productivity measure used (GDP, gross value added, average remuneration, minimal wage, total employment costs); valuation of non-market losses (informal care and unpaid housekeeping work); and inclusion of intangible costs associated with pain and suffering which are particularly difficult to estimate. Moreover, the magnitude of economic burden in some cost categories depends on the institutional characteristics of an economy; e.g. an increase of retirement age in a country elevates productivity costs due to morality at a certain working age. Nevertheless, having these limitations in mind, we think that there is room for improving the comparability of estimates from different studies. Specifically, we recommend presenting the costs categories in values which are neutral to the economic power or population size of a country/region. In small and less developed countries the absolute costs of a disease are obviously lower than in larger and wealthier ones even if the relative economic burden of the disease is greater in the former. By using relative costs we could make an easy step forward in gaining more international/interregional comparability of results. Relating the costs to GDP seems to be most obvious option, as this measure is extensively used and widely understandable in general public. Of the reviewed studies on productivity losses in BC [15, 16, 18–23] there is only one that presents costs in values relative to GDP; according to the estimates from Korea the total costs (both direct and indirect) of BC in the country in 2007–2010 period ranged from 0.06% to 0.09% of GDP [19]. The shares for particular cost categories are not reported in the Korean study, still this way of data presentation is a step forward comparing to other research. We recommend to use the same approach for all cost categories included in the analysis allowing for easier and more detailed comparisons across countries/regions. Yet, if a disease or a cost category yields comparatively low costs relative to GDP (like caregiver’s presenteeism here which accounts for 0.0001% of GDP across the whole period) we recommend to use a multiplicity of per capita GDP. Using this approach caregivers’ presenteeism accounted for 37 and 45 per capita GDP in 2010 and 2014, respectively. As these values show, the magnitude of this cost category is low and in such a case by using multiplicity of per capita GDP we obtain an appealing and comprehensible measure. Obviously, expressing the cost categories relative to GDP could not overcome other abovementioned problems of results comparability, still it seems to be a step forward.

Before concluding we shall acknowledge the limitations of our estimates. Firstly, the study used a variety of sources and in some cases in the absence of real data (e.g. absenteeism in farmers population or 5-year prevalence of BC for most years) we had to rely on approximated values. This could potentially bias the results and they need to be interpreted with caution; still, a similar issue arises in most studies that aim to estimate indirect costs of diseases. Secondly, we had to make some methodological choices, particularly on the method of costs estimation and on the productivity measure used. Applying human capital method is subject to criticism in health economics research [40, 41]. Principally, the method may over-estimate the real burden of the disease because it is built on an assumption that a sick person cannot be replaced by an unemployed one. Moreover, it does not take economy’s fluctuations into consideration and implicitly assumes that there is no unemployment, while those working are fully efficient [24]. Despite these drawbacks HCM is the most commonly used method for estimating productivity losses attributable to various diseases because it has strong economic foundations and tradition [15]. Moreover, the alternative of friction costs method poses other methodological challenges making it more difficult in practice and is not well grounded in economic theory (for the review of both methods and their criticism see [42]). Summing up, HCM seems to be a reasonable choice; though, it needs to be stressed that it estimates potential or maximum losses. Considering productivity measure, we used per worker GDP which is also questioned and several alternatives are used in other studies (e.g. gross value added); in this case, we believe that a GDP-based measure is appealing for general public, making the results more comprehensible. Thirdly, although we were able to estimate the losses associated to presenteeism, the scope of productivity decrease due to BC was approximated by the values estimated for other countries (presenteeism of the sick) or for all cancers (caregivers’ presenteeism). This caveat has to be kept in mind when interpreting the magnitude of reduced efficiency at work. Finally, because of data unavailability the analysis did not consider the value of housekeeping activities undone due to BC which constitute an important category of losses as a study from Flanders shows (8% of total BC costs) [20].

## Conclusions

In conclusion, we estimated the productivity losses and public finance burden attributable to breast cancer in Poland in the years 2010–2014. The indirect cost of the disease is substantial and accounted for around 0.162–0.171% of GDP throughout the period. BC was also a sizeable burden for the public finance contributing both to increased expenditure on social insurance benefits and diminishing tax revenues. These economic losses might be confronted through several actions at each stage of BC management, namely, prevention and screening of the disease, early-stage treatment and provision of care for BC survivors. Bearing in mind that the incidence of BC among women at working age in Poland is growing and regarding the anticipated decrease of labour supply in the country the actions aimed at BC patients’ recovery seem to be not only crucial for their well-being but also for the economy’s prosperity. Following this reasoning the costs of BC treatment may well be considered as an investment and the estimates provided by this analysis can be used to determine priorities and to inform public policy choices.

## Abbreviations

BC: Breast cancer
CIT: Corporate income tax
GDP: Gross domestic product
HCM: Human capital method
KRUS: Agricultural Social Insurance Fund (*Kasa Rolniczego Ubezpieczenia Społecznego*)
PIT: Personal income tax
US: United States
VAT: Value added tax
ZUS: Social Insurance Institution (*Zakład Ubezpieczeń Społecznych*)

## Footnotes

1 The age group 15–59 years does not strictly correspond to the productive age of women in Poland (18–59 years); the age interval used is determined by the way that the Polish National Cancer Registry reports the age-specific data. Nevertheless, accounting for the age distribution of BC, we can expect that both populations are practically the same.

2 Following [24] we assumed that a person partially incapable to work is able to work with 0.25 productivity of a healthy person; this assumption is based on the fact that the value of benefit received in this case is 0.75 of the benefit received by a person completely incapable to work.

3 In 2014 these four taxes contributed to 89% of state budget revenues.

4 Social pension is a benefit which is payable to an adult who has been recognised as completely incapable of work due to impairment of body functions which occurred before reaching the age of 18 years.
